# Contrasting drivers of belowground nitrogen cycling in a montane grassland exposed to a multifactorial global change experiment with elevated CO_2_, warming, and drought

**DOI:** 10.1111/gcb.16035

**Published:** 2022-01-10

**Authors:** Tania L. Maxwell, Alberto Canarini, Ivana Bogdanovic, Theresa Böckle, Victoria Martin, Lisa Noll, Judith Prommer, Joana Séneca, Eva Simon, Hans‐Peter Piepho, Markus Herndl, Erich M. Pötsch, Christina Kaiser, Andreas Richter, Michael Bahn, Wolfgang Wanek

**Affiliations:** ^1^ Division of Terrestrial Ecosystem Research Department of Microbiology and Ecosystem Science Center of Microbiology and Environmental Systems Science Vienna Austria; ^2^ INRAE Bordeaux Sciences Agro ISPA Villenave d'Ornon France; ^3^ Institute of Crop Science University of Hohenheim Stuttgart Germany; ^4^ Agricultural Research and Education Centre Raumberg‐Gumpenstein Irdning‐Donnersbachtal Austria; ^5^ Department of Ecology University of Innsbruck Innsbruck Austria

**Keywords:** amino acid consumption, climate warming, drought, elevated CO_2_, protein depolymerization, soil nitrogen cycling, T‐FACE

## Abstract

Depolymerization of high‐molecular weight organic nitrogen (N) represents the major bottleneck of soil N cycling and yet is poorly understood compared to the subsequent inorganic N processes. Given the importance of organic N cycling and the rise of global change, we investigated the responses of soil protein depolymerization and microbial amino acid consumption to increased temperature, elevated atmospheric CO_2_, and drought. The study was conducted in a global change facility in a managed montane grassland in Austria, where elevated CO_2_ (eCO_2_) and elevated temperature (eT) were stimulated for 4 years, and were combined with a drought event. Gross protein depolymerization and microbial amino acid consumption rates (alongside with gross organic N mineralization and nitrification) were measured using ^15^N isotope pool dilution techniques. Whereas eCO_2_ showed no individual effect, eT had distinct effects which were modulated by season, with a negative effect of eT on soil organic N process rates in spring, neutral effects in summer, and positive effects in fall. We attribute this to a combination of changes in substrate availability and seasonal temperature changes. Drought led to a doubling of organic N process rates, which returned to rates found under ambient conditions within 3 months after rewetting. Notably, we observed a shift in the control of soil protein depolymerization, from plant substrate controls under continuous environmental change drivers (eT and eCO_2_) to controls *via* microbial turnover and soil organic N availability under the pulse disturbance (drought). To the best of our knowledge, this is the first study which analyzed the individual versus combined effects of multiple global change factors and of seasonality on soil organic N processes and thereby strongly contributes to our understanding of terrestrial N cycling in a future world.

## Graphical abstract

Organic nitrogen (N) cycling is recognized as the major process controlling the overall soil N cycle, yet few studies investigated global change responses of soil organic N breakdown. We focused on the interactive effects of multiple global change drivers (warming × CO_2_ × drought) on soil protein depolymerization and microbial amino acid consumption. We found a shift in controls of these processes, from plant controls under warming and elevated CO_2_, to microbial turnover and soil organic N availability under push disturbances (drought). Seasonality modulated the effects of warming by changing the factors limiting soil organic N cycling.

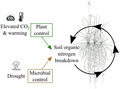

## 1 INTRODUCTION

Nitrogen (N) is one of the most essential elements across terrestrial ecosystems. It is a macronutrient that constrains growth and activity of all living organisms, including plants and soil microorganisms. It regulates soil organic matter decomposition, and can influence the ecosystem response to global climate change (Brevik, 2012; Pugnaire et al., 2019). For example, N limitation has been found to strengthen the stimulatory effects of elevated CO_2_ on soil respiration (Gao et al., 2020) and to constrain the CO_2_ fertilization effect on plant productivity (Terrer et al., 2019). In soil systems, N is mainly present in high‐molecular weight organic N forms (HMW‐ON), that is polymeric compounds, which need to be converted into smaller oligomers or monomers (i.e., depolymerized) to become bioavailable to plants and microorganisms (Figure S1). These low‐molecular weight (LMW) organic N compounds can then be mineralized by microbes or directly be taken up by plants. While classically N mineralization was considered to be the limiting step in the soil N cycle (Odum, 1966; Vitousek, 1982), more recent evidence has shown that depolymerization of HMW‐ON polymers (such as proteins, peptidoglycan, and chitin) is the bottleneck of the soil N cycle (Hu et al., 2020; Jan et al., 2009; Schimel & Bennett, 2004; Wanek et al., 2010). Because over 50% of total soil N is represented by peptide structures (Schulten & Schnitzer, 1998) and contributes approximately 90% to total N in plant litter and microbial residues, depolymerization of proteins to oligopeptides and free amino acids drives the soil N cycle and determines the amount of N available to plants and microbes (Jan et al., 2009; Jones et al., 2002). Despite the importance of these processes, we have little knowledge on how they respond to environmental change.

Globally, the air temperature is expected to rise by 2–4°C within this century, and atmospheric CO_2_ is predicted to increase by 100–300 ppm (IPCC, 2014). Climate models also predict stronger and more frequent drought periods in many regions, including montane grasslands that have traditionally experienced year‐round moist conditions (Gobiet et al., 2014). Terrestrial N cycling has been altered by global change, including increasing atmospheric CO_2_, climate warming, and drought, which can lead to potential feedbacks to climate change (Zaehle, 2013; Zaehle et al., 2010). However, most studies have focused on the inorganic part of the soil N cycle, such as organic N mineralization and nitrification (Bai et al., 2013; Borken & Matzner, 2009; Hartmann et al., 2013; Rustad et al., 2001; Séneca et al., 2020). Gross rates of protein depolymerization have only been reported in a handful of studies (Hu et al., 2020; Mooshammer et al., 2012; Noll, Zhang, & Wanek, 2019; Prommer et al., 2014; Wanek et al., 2010; Wild et al., 2013; Wild, Alves, et al., 2018), and only three of these have studied its response to global change drivers in a field setting (Andresen et al., 2015; Fuchslueger et al., 2019; Wild, Ambus, et al., 2018). In one study, 8 years of environmental change manipulation (warming, elevated atmospheric CO_2_, and drought), as single treatments or combined, did not affect gross rates of protein depolymerization in heathland soils in a late autumn sampling campaign (Wild, Ambus, et al., 2018). While summer drought has been found to increase gross protein depolymerization in an extensively managed as well as in an abandoned subalpine grassland (Fuchslueger et al., 2019), it was found to have a negative effect on gross protein depolymerization in a native heathland (Andresen et al., 2015). However, little is known about how different global change drivers influence soil organic N processes throughout different seasons.

The release of N from proteins in soil is mediated by multiple factors, which might affect responses to environmental change. Extracellular peptidases and proteases, which are a highly diverse class of enzymes, catalyze the breakdown of proteins and are released into the soil by microbial decomposers and by plant roots (Nguyen et al., 2019; Vranova et al., 2013). Microbial community size and structure, soil C and N availability, along with a range of environmental factors, strongly modulate their activity (Brzostek & Finzi, 2011; Geisseler & Horwath, 2008; Noll, Zhang, & Wanek, 2019). A meta‐analysis found that warming and drought adversely affected potential protease activity across 16 global change experiments, and the differential responses were caused by differences in soil moisture (Brzostek et al., 2012). In contrast, in a recent meta‐analysis, extracellular enzymes involved in the soil N cycle were relatively unresponsive to global change treatments (Xiao et al., 2018). Climate change effects may be constrained by the reduced availability of proteins as substrates for proteolytic enzymes, through processes such as their occlusion in microaggregates or the sorption to soil minerals (Noll, Zhang, & Wanek, 2019). Furthermore, their responses may change with seasonal changes in climate and vegetation phenology. Indeed, recent evidence has shown that seasonality can modulate the effects of warming on extracellular enzymes and on microbial growth (Simon et al., 2020; Zuccarini et al., 2020).

Our overall objective was to investigate the effects of different global change factors on gross rates of soil organic N processes across the growing season. Specifically, we tested the effects of increased atmospheric CO_2_ (eCO_2_), elevated temperature (eT), and their interaction, in a unique multifactorial experimental design in a managed montane grassland (i.e., receives equal fertilization in each plot, after each aboveground cut) in the Austrian Alps (Piepho et al., 2017). Furthermore, we tested for the effects of summer drought on additional plots, which were exposed to ambient or future (eT + eCO_2_) environmental conditions. We carried out three sampling campaigns, in May, July, and October 2017, to identify the role of seasonality in modulating climate change effects. The effects of eCO_2_ and eT were evaluated at three levels each (including ambient conditions), to investigate possible nonlinear responses.

We tested the following hypotheses: (H_1_) eCO_2_ would not have a significant effect on gross protein depolymerization or microbial amino acid consumption rates, because all plots of the managed grassland studied were fertilized, irrespective of treatment, following common practice of managed grasslands. Fertilization is assumed to buffer a potential increase in plant N demand and in soil N mining with eCO_2_. (H_2_) eT would lead to an increase in protein depolymerization and amino acid consumption rates, but only during the early growing season. In general, eTs increase soil enzyme activity and net N mineralization and nitrification (Bai et al., 2013). However, reduced protein substrate availability as the growing season progresses will reduce the temperature effect (Brzostek & Finzi, 2011). (H_3_) Drought would have a negative effect on protein depolymerization and amino acid consumption rates, because proteolytic enzyme activity is likely to decrease under water‐limited conditions (Homyak et al., 2017). Finally, (H_4_) we expected no effect of eT × eCO_2_, due to the absence of an eCO_2_ effect. We also expected the effect of drought to be less negative in the “future scenario” (eT + eCO_2_) plots, where organic N processes are stimulated by eT.

## 2 MATERIALS AND METHODS

### 2.1 Site description and sample collection

The study site is located at the Agricultural Research and Education Center (AREC) in Raumberg‐Gumpenstein, a managed montane grassland in the Austrian Alps, Styria, Austria. (47°29'38"N, 14°06'03"E). The climatic site conditions with a mean annual temperature of 8.5°C and a mean annual precipitation of 1009 mm (average 1991–2020) are representative for a larger geographic area of montane grasslands in Central Europe. The soil type is a Cambisol (World Reference Base classification) with a loamy sand texture and a pH of ~5.5. Before establishment of the global change experiment (ClimGrass), a typical grassland mixture was sown in an area of homogeneous soils in 2007, comprising the grass species *Arrhenatherum elatius* L., *Dactylis glomerata* L., *Poa pratensis* L., *Alopecurus pratensis* L., *Festuca rubra* L., *Trisetum flavescens* L., and *Festuca pratensis* L., and the legumes *Lotus corniculatus* L. and *Trifolium repens* L. The ClimGrass project entails 54 plots with a T‐FACE (Temperature – Free Air Carbon dioxide Enrichment) setup, put into full operation in 2014, to manipulate temperature and CO_2_ at three levels each including ambient conditions (Figure S2; described in Piepho et al., 2017). Warming is performed full‐time all year‐round (day and night), unless the snow cover exceeds a height of 10 cm, at which point the system is turned off (it is reinitiated when the snow depth reaches <10 cm again). The CO_2_ fumigation takes place only during the growing season (begin of April to the end of November) and during the day as soon as global radiation is above 50 W m^−2^. All plots regularly received the same amount of mineral fertilizer to replace nutrients removed by the harvests (spring: 30 kg N, 32.5 kg P, 85 kg K; after first harvest: 30 kg N; after second harvest: 30 kg N).

**FIGURE 1:**
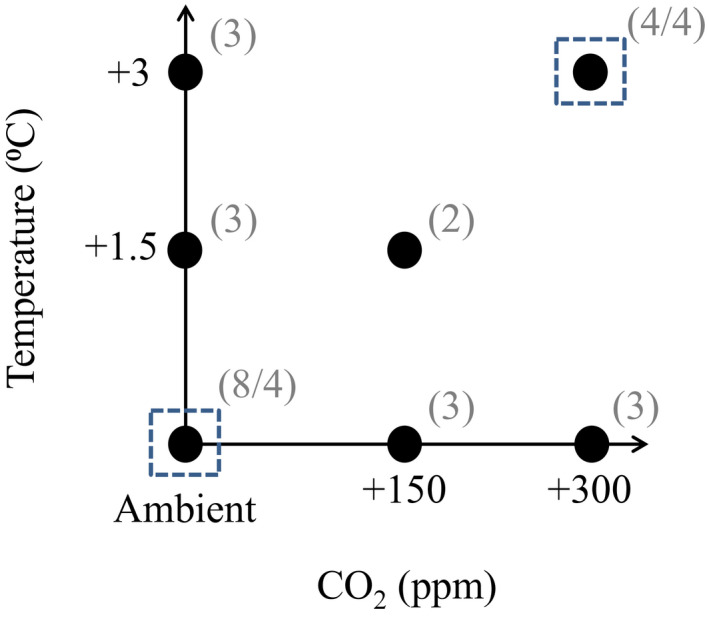
Graphic representation of the plots from which samples were collected and analyzed (*detailed in* Simon et al., 2020). The figure illustrates the different combinations of three temperature levels, three CO_2_ levels, and drought (*blue* dashed squares). The gray numbers in brackets represent the number of sample replicates (plots) per treatment, and the number after the slash refers to the available replicates for the drought and rewetting treatment. The setup follows the response surface approach, which includes seven of nine possible treatment combinations (Piepho et al., 2017)

For this project, 34 plots in a factorial design with varying manipulations of temperature (ambient, +1.5, and +3.0°C), CO_2_ (ambient, +150, and +300 ppm), and drought were sampled (Figure 1). The overall design strategy takes account of budget constraints imposing limitations on the number of plots with eT and CO_2_ levels, minimizing the number of replicates necessary. The approach is based on polynomial regression models (a surface response approach, Piepho et al., 2017) and is focused on efficient estimation of interactions between the two treatment factors. Previously reported analyses demonstrated the overall suitability of the proposed design to analyze nonlinear interactions of two or more global change factors (Piepho et al., 2017). In 2017, four plots exposed to ambient conditions and three plots exposed to +3.0°C and +300 ppm CO_2_ were subjected to a summer drought event using automated rainout shelters. The drought treatment started on May 23 and lasted until July 26. A scheduled rewetting with 40 mm of previously collected rainwater was performed on July 27, after which the shelters were deactivated to analyze the effects of drought recovery (details in Simon et al., 2020). The “drought” treatment hereafter refers to the soil sampling at the end of the drought, and the “recovery” treatment refers to the soil sampling 3 months after the scheduled rewetting (detailed dates below).

Three soil sampling campaigns were conducted for the seasonal analysis, each directly on the day of the plant harvests, that is on May 30–31, July 25–26, and October 3–4 in 2017. Fresh aboveground plant biomass of the plot harvests was weighed, a well‐mixed aliquot dried at 50°C for 48 h, passed through a 1‐mm screen, and a subsample finally dried at 105°C (determination of residual water), to estimate aboveground biomass and aboveground net primary production (ANPP). To test the combinations of different climate scenarios on fine root turnover, ingrowth cores were installed in 37 of the plots, ensuring an even distribution among all treatments. One collection of soil cores, representing the status quo before the treatments, was harvested, separated into three soil depths (0–10 cm, 10–20 cm, and 20–30 cm), and stored at −18°C, before collecting and washing the fine roots. The ingrowth cores (wire cover with a diameter of 4 cm, 30 cm length, and a mesh size of 0.36 cm) were filled with root‐free, sieved soil from the ClimGrass‐site and exchanged three times during the growing season in 2017. All root samples were sorted by depth into three categories: 0–10 cm, 10–20 cm, and 20–30 cm (Sarah Helena Geiger, MSc thesis, Univ. Innsbruck, Austria). At the same time as the plant cuts were performed, the fine root biomass was collected to estimate fine root biomass production (belowground net primary production) and root turnover (standing biomass divided by root ingrowth from previously installed root ingrowth cores). From each plot, a minimum of five soil samples were collected to meet soil requirements for all analyses using a soil corer of 2 cm diameter to 10 cm soil depth. The samples were then pooled, fresh masses weighed, and soils sieved through a 2‐mm mesh. Fine roots were picked, washed, and dried to estimate fine root biomass. Aliquots of fresh sieved soil were weighed and dried (85°C, 48 h) to calculate the fresh to dry weight ratio and the soil water content. Other aliquots were used to measure the soil amino acid content, gross rates of protein depolymerization and microbial amino acid consumption rates (and of organic N mineralization and nitrification), microbial biomass carbon and nitrogen, and potential enzyme activities (dataset: Maxwell et al., 2021).

### 2.2 Amino acid and ammonium quantification

Total free amino acid concentrations were measured in order to quantify the environmental change effects on this labile organic N pool as well as to calculate maximum tracer addition rates for ^15^N isotope pool dilution (IPD) assays of protein depolymerization and microbial amino acid consumption. An aliquot (2 g fresh weight) per soil sample was extracted with 15 ml 1 M KCl for both amino acid and ammonium quantification. Amino acids were measured by fluorimetric determination: a mix of *o*‐phthaldialdehyde and 3‐mercaptopropionic acid (OPAME) was added to the samples, yielding a fluorigenic product that was measured at an excitation wavelength of 340 nm and an emission wavelength of 450 nm (Jones et al., 2002; Prommer et al., 2014) with a fluorimeter (Tecan Infinite 200). In order to correct for ammonium fluorescence, its concentration was measured in the same soil extracts by colorimetric determination (Hood‐Nowotny et al., 2010; Kandeler & Gerber, 1988). By using concentration standards and the resultant calibration curves, the measured fluorescence of the sample, the original soil fresh weights, and the fresh to dry weight ratios, soil ammonium and amino acid concentrations were calculated. Fluorescence quenching was corrected for via a spiking standard (amino acid mix) added to all samples.

### 2.3 Isotope pool dilutions

#### 2.3.1 Gross protein depolymerization

The total free amino acid pool sizes in the fresh soils were measured 1 day prior to the IPD assays to calculate the ^15^N substrate addition rates. Approximately 20% of the native amino acid‐N was added as a ^15^N‐labeled amino acid mix (18 algal amino acid mixture, 96–98 atom% ^15^N, Cambridge Isotope Laboratories) to the target amino acid pool (Wanek et al., 2010; Wild et al., 2013) for the IPD assay. Two aliquots (2 g fresh weight) per sample were run at in situ field temperatures (Table S1): one was stopped 15 min after tracer addition and the other after 60 min by the addition of 10 ml cold (4°C) 1 M KCl, effectively halting enzymatic activity and extracting free amino acids (Hu et al., 2017). The suspensions were shaken for 30 min, filtered through ash‐free cellulose paper, and subsequently stored in a freezer at −20°C until further analysis.

Prior to quantifying the ^15^N:^14^N ratios and the concentrations of free amino acids in the thawed extracts, NH_4_^+^ had to be removed from the extracts by microdiffusion, as it interferes with the conversion of α‐amino groups (‐NH_2_) of amino acids to nitrite (Noll, Zhang, Zheng, et al., 2019). The solution pH was increased to >9.5 by MgO addition to shift the NH_4_^+^/NH_3_ equilibrium to the volatile NH_3_, which was collected by acid traps made from Teflon tape and filter paper, acidified by addition of 4 µl 2.5 M KHSO_4_ (Lachouani et al., 2010). After 2 days on an orbital shaker at room temperature, the acid traps were removed and discarded.

Free amino acids in the ammonium‐free extracts were then converted to N_2_O gas which was analyzed by purge‐and‐trap isotope ratio mass spectrometry (PT‐IRMS) for the isotopic composition and concentration of the amino acids (Noll, Zhang, Zheng, et al., 2019; Zhang & Altabet, 2008). The α‐amino group (‐NH_2_) of amino acids was first released as NH_3_ by Strecker degradation. The resulting NH_3_ was further oxidized to NO_2_^−^ with sodium hypobromite under alkaline conditions (pH >12) and the reaction quenched by addition of an excess of sodium arsenite. In the final reduction step, NO_2_^−^ was converted to N_2_O by a buffered sodium azide solution (NaN_3_) and the N_2_O analyzed by PT‐IRMS with a cryo‐focusing unit (GasBench II coupled to Delta V Advantage, Thermo Fisher Scientific). This allowed for the sensitive determination of the concentration and the at%^15^N of free amino acids in the soil extracts. The measured amino acid concentrations were compared to the results obtained by the fluorimetric method, allowing to detect potential outliers in the PT‐IRMS measurements.

### 2.4 Gross N mineralization and nitrification

Using the previously photometrically determined NH_4_^+^ and NO_3_^−^ pool sizes, we calculated the concentration of the ^15^NH_4_Cl and K^15^NO_3_ (98 at%) tracer solutions, to approximately add 20% of the target pool in ^15^N‐labeled form. The tracer was added to 2–3 g of duplicate fresh soil samples, which were incubated at in situ field temperature (Table S1): one aliquot was stopped 4 h after tracer addition and the other after 24 h by the addition of cold (4°C) 1 M KCl (1:7.5 w‐v). The samples were then shaken on an orbital shaker for 30 min (150 rpm) and then filtered through ash‐free cellulose filters. The mineralization extracts were prepared using the microdiffusion method (as described in the protein depolymerization protocol above), followed by the measurement of ^15^/^14^N isotope ratio by elemental analyzer (EA)‐IRMS (EA1110 analyzer coupled via ConFlo III interface to a Delta^PLU^S IRMS, Thermo Fisher Scientific). Concentrations and ^15^/^14^N isotope ratios of NO_3_^−^ in the 1 M KCl extracts were determined by converting NO_3_^−^ to NO_2_^−^ with vanadium (III) chloride (VCl_3_) under acidic conditions and further reduction of NO_2_^−^ to N_2_O by sodium azide (NaN_3_) (Lachouani et al., 2010). Concentrations and ^14^/^15^N isotope ratios of the resulting N_2_O were determined by PT‐IRMS with a cryo‐focusing unit (GasBench II coupled to Delta V Advantage, Thermo Fisher Scientific). Parts of the data of gross rates of inorganic N cycling processes (reduced treatments, and for the July harvest only) have previously been published and analyzed (Séneca et al., 2020).

### 2.5 Isotope pool dilution calculations

#### 2.5.1 Gross nitrogen transformation rates

The fluxes into (influx, equivalent to protein depolymerization, mineralization, nitrification) and out of the target pools (free amino acids, ammonium, nitrate) (efflux, i.e., microbial amino acid consumption, ammonium consumption, nitrate consumption, the latter two processes not being shown here) between the two time points were calculated using the isotope mass balance equations developed by Kirkham and Bartholomew (1954):

(1) $$\text{Gross influx}\;\left(\text{GI}\right)\;\text{rate}\left[\mu g\;{\text{Ng}}^{‐1}\;\text{dm}\;d^{‐1}\right]=\frac{C_{t2}‐C_{t1}}{\left(t_{2}‐t_{1}\right)/60/24}\times \frac{\ln\left(\frac{{\text{APE}}_{t1}}{{\text{APE}}_{t2}}\right)}{\ln\left(\frac{C_{t2}}{C_{t1}}\right)}$$

(2) Gross efflux(GE)rateμgNg‐1dmd‐1=Ct1‐Ct2t2‐t1t2‐t160/2460/24×1+lnAPEt2APEt1lnCt2Ct1

where t_1_ and t_2_ are the two time points (min) when soil incubations were stopped, C_t1_ and C_t2_ represent the total amino acid, ammonium or nitrate concentrations (^14^N+^15^N) (μg N g^−1^ dry mass), and APE is ^15^N atom% excess (measured atom%^15^N sample – atom%^15^N background) of the respective pools (Figure S3).

### 2.6 Mean residence times

The mean residence time of free amino acids in soils was calculated by dividing the free amino acid contents (pool size) by the average of gross influx (GI) and gross efflux (GE) rates. As GI and GE rates are per day, the average mean residence time was calculated in hours as follows:

(3) $$\text{Mean residence time}\left[h\right]=\frac{\text{Pool size}\left[\mu g\;{\text{Ng}}^{‐1}\;\text{dm}\right]}{\text{Average}\left(\text{GI}+\text{GE}\right)\left[\mu g\;{\text{Ng}}^{‐1}\;\text{dm}\;d^{‐1}\right]}\times 24\left[h\;d^{‐1}\right]$$

### 2.7 Complementary soil analyses

Total soil organic C and soil total N were measured in aliquots of ball‐milled oven‐dried soil by EA‐IRMS (EA1110 coupled by ConFlo III to Delta^PLUS^ IRMS, Thermo Scientific). Dissolved organic C and N pools were measured in 1 M KCl extracts (1:7.5 w:v, for 1 h) after extracting aliquots of 4 g field‐moist soil for 60 min, filtration through ash‐free cellulose filters, and storage at −20°C until analysis. Dissolved organic C (DOC) and total dissolved N (TDN) were analyzed by a TOC/TN analyzer (TOC‐VCPH/TNM‐1, Shimadzu, Austria). Nitrate concentrations were measured in the same extracts by colorimetric assays as described by Hood‐Nowotny et al. (2010). Dissolved organic N (DON) was calculated as TDN minus ammonium and nitrate. Microbial biomass C and N were determined using the chloroform‐fumigation extraction method (Brookes et al., 1985). Soils were fumigated with chloroform for 48 h and extracted (1:7.5 (w:v)) with 1 M KCl, and DOC and TDN measured as mentioned above. Leucine‐amino peptidase (LAP) and tyrosine‐amino peptidase (TAP) activities were measured fluorimetrically with l‐leucine‐7‐amido‐4‐methyl coumarin (AMC‐leucine, 1 mM) and l‐tyrosine‐7‐amido‐4‐methyl coumarin (AMC‐tyrosine, 1 mM) in Na‐acetate buffered (100 mM, pH 5.5) soil slurries using a microtiter plate assay (Kaiser et al., 2010). The samples were run in five technical replicates and measured every 30 min for 2 h. Fluorescence was measured with a InfiniteR M200 fluorimeter (TECAN, Austria) at an excitation wavelength of 365 nm and an emission wavelength of 450 nm, and corrected for sample blank and quenching prior to calculations of released AMC concentration. Microbial growth, turnover time, and nitrogen use efficiency (NUE) were measured according to ^18^O incorporation into soil microbial DNA from ^18^O‐labeled soil water, and ^18^O isotope and DNA analysis performed as published previously (Zhang et al., 2019; Zheng et al., 2019). Gross N mineralization, ammonification, and nitrification were determined using ^15^N IPD measurements (Wanek et al., 2010; Zhang et al., 2019).

Soil microbial biomass of major microbial taxa were estimated by extracting phospholipid fatty acids (PLFAs) from freeze‐dried soil samples with a high throughput method (Buyer & Sasser, 2012), with some modifications. Total lipids were extracted from soils using a chloroform/methanol/citric acid buffer mixture and fractionated by solid‐phase extraction on silica columns. The neutral lipid fatty acid (NLFA) fraction was collected by eluting the cartridges with chloroform, while the PLFA fraction was collected by eluting the columns with a 5:5:1 chloroform:methanol:water mixture. After addition of an internal standard (19:0), NLFAs and PLFAs were converted to fatty acid methyl esters by transesterification. Samples were analyzed for identification and quantification using a GC (7890B GC System; Agilent, Santa Clara, CA, USA) connected to a TOF/MS (Pegasus HT; LECO Corporation). Samples were injected in splitless mode (injector temperature 220°C) and separated on a DB5 column (60 m 0.25 mm × 0.25 μm; Agilent) with 1.5 mL He min^−1^ as the carrier gas (GC program: 1 min at 80°C, 30°C min^−1^ to 150°C, 1 min at 150°C, 2°C min^−1^ to 200°C, 4°C min^−1^ to 230°C, 15 min at 230°C, 30°C min^−1^ to 290°C and 5 min at 290°C). FAMEs were identified using mixtures of bacterial and fungal FAMEs (Bacterial Acid Methyl Ester CP Mixture (Matreya LLC) and the 37 Comp. FAME Mix (Supelco)). FAMEs were quantified against the internal standard (19:0). We used the PLFA markers 18:1ω9 and 18:2ω6,9 to estimate fungal biomass, and 16:1ω5 for arbuscular mycorrhizal fungi. However, while 16:1ω5 is a marker often used for arbuscular mycorrhizal fungi, it can also originate from gram‐negative bacteria (Frostegård et al., 2011). Therefore, the NLFA 16:1ω5 was also used to quantify arbuscular mycorrhizal fungi, as this biomarker is more specific for this microbial group. The sum of PLFA i15:0, a15:0, i16:0, i17:0, and a17:0 was used as gram‐positive bacterial markers, and 16:1ω7, 18:1ω7, cy17:0, and cy19:0 as gram‐negative bacterial markers (Quideau et al., 2016). The sum of 10Me16:0, 10Me17:0, and 10Me18:0 was used as marker for Actinobacteria (Brennan, 1988; Quideau et al., 2016). Gram‐positive, gram‐negative, and Actinobacterial markers were summed to give total bacterial PLFAs. The remaining peaks, including the PLFA general markers 16:0, 17:0, and 18:0, which cannot be assigned to bacterial nor fungi exclusively, and peaks with double bond position, which could not be chromatographically resolved, were assigned to the general PLFA marker group (Quideau et al., 2016).

### 2.8 Statistical analyses

Statistical analyses were performed with R 3.1.3 (R Development Core Team), and graphs were generated using the R “ggplot2” package (Wickham, 2016). Supplementary graphs were generated using Sigma Plot 12.0 and the 3D plots were generated with the R package “rsm” (Lenth, 2009). The experiment consists of two different approaches, a response surface approach, including the three levels of atmospheric temperature and CO_2_ concentration manipulation (including ambient conditions), and an ANOVA design, including ambient and a “future environmental scenario” (combining highest levels of eCO_2_ and eT) ± drought. We also applied a correlation approach to study the main variables explaining the protein depolymerization rates.

### 2.9 Response surface regression approach

To test effects of eT and eCO_2_ on the N pools and processes (protein depolymerization, microbial amino acid consumption, mean residence time of amino acids, organic N mineralization, and nitrification) across seasons, we first used a quadratic generalized least squares (GLS) model with the R package “nlme” (Pinheiro & Bates, 2020). We accounted for autocorrelation between the sampling dates (R function *corr* = corrCompSymm) and used an additional *weights* function to allow for heterogeneous variance between sampling dates, similar to Simon et al. (2020). We included all the levels of temperature and CO_2_ (ambient, +1.5°C, +3°C; and ambient, +150 ppm, +300 ppm), enabling us to test both first and second‐order factors in order to evaluate possible nonlinear responses to multiple levels of temperature and CO_2_ (Piepho et al., 2017). We then reduced the model by deleting each of the nonsignificant variables tested to observe the marginality principle (Piepho & Edmondson, 2018), and finally included only the significant terms for the analysis of variance of these models in Table 1. Normal distribution and homogeneity of variance were checked by inspecting plots of standardized residuals versus predicted values, frequency histograms, and Q–Q plots, as well as applying the Shapiro and Levene tests, respectively. Mineralization rates, soil free amino acids contents, and mean residence time data were log‐transformed before statistical analyses to satisfy the assumption of normality. The significance threshold was set to α = 0.05 for all performed tests.

**TABLE 1:** Results of the generalized least squares (GLS) models to test for the effects of global change drivers on free amino acids, protein depolymerization, amino acid consumption, the mean residence time of amino acids, mineralization, and nitrification rates. An overarching model included the effects of season, elevated CO_2_, and elevated temperature and their interaction, along with CO_2_ and temperature as quadratic terms, and these terms in interaction with season. This model was reduced for each of the studied variables following the marginality principle (Piepho & Edmondson, 2018). Significant results are in bold (*p* < .05)

	Free amino acids		Protein depolymerization		Amino acid consumption		Mean residence time		Mineralization		Nitrification	
	*F*‐value	*p*‐value	*F*‐value	*p*‐value	*F*‐value	*p*‐value	*F*‐value	*p*‐value	*F*‐value	*p*‐value	*F*‐value	*p*‐value
Season	**249.38**	**<.0001**	**27.18**	**<.0001**	**48.47**	**<.0001**	**62.58**	**<.0001**	0.13	.7247	**58.81**	**<.0001**
CO_2_	0.71	.4023	–	–	**4.90**	.**0300**	**3.37**	.0705	0.47	.4952	–	–
Temp	0.63	.4312	3.56	.0630	0.90	.3467	**4.57**	.**0359**	**4.01**	.**0490**	–	–
Temp^2^	**6.78**	.**0112**	–	–	–	–	–	–	–	–	–	–
CO_2_^2^	–	–	–	–	1.43	.2352	–	–	–	–	–	–
Season × CO_2_	–	–	–	–	–	–	–	–	–	–	–	–
Season × temp	–	–	**28.17**	**<.0001**	**13.54**	.**0004**	**22.862**	**<.0001**	–	–	–	–
CO_2_ × temp	**6.82**	.**0110**	–	–	–	–	–	–	**12.973**	.**0006**	–	–
Season × temp^2^	–	–	–	–	–	–	–	–	–	–	–	–
Season × CO_2_^2^	–	–	–	–	–	–	–	–	0.4097	.5241	–	–
Season × CO_2_ × temp	–	–	–	–	–	–	–	–	–	–	–	–

### 2.10 ANOVA approach

Drought events were simulated in the ambient and in the “future environmental scenario” (combined +3°C and +300 ppm) plots (Figure 1). The sampling in May represents a control to test the absence of preexisting differences between the untreated plots and their replicates for which a drought would be put into effect (from late May to late July). The July and October sampling dates represent the drought and recovery periods, respectively. The pools and processes were analyzed separately for each season using two‐way ANOVAs: effects of climate treatment (ambient *vs*. “future”) and drought or recovery after drought (ambient *vs*. drought) were assessed as main factors, as well as their interaction. To check for significant differences between treatments, Tukey's HSD tests were performed for each season.

### 2.11 Correlations with protein depolymerization rates

We ran repeated measures correlation analyses to assess the relationship between gross protein depolymerization and other edaphic and vegetation parameters, including plant and microbial descriptors, N pool sizes, and enzyme activities. This was done using the “rmcorr” package (Bakdash & Marusich, 2017), which accounts for nonindependence among observations, using analysis of covariance (ANCOVA) to statistically adjust for interindividual variability. This was done in order not to violate the assumption of independence due to the repeated measures in our experimental design. *P*‐values in the “rmcorr” package were obtained by a bootstrapping approach (Bakdash & Marusich, 2017). The normality and homoscedasticity assumptions were verified visually (as described above) and data were log‐transformed when necessary to meet the model assumptions. We performed three separate analyses, that is for (i) the effect of temperature and CO_2_ level across seasons (“eT eCO_2_ subset”), which includes the ambient, as well as warmed (+1.5°C, +3°C) and elevated CO_2_ combinations (+150 ppm, +300 ppm) across all three seasons. Then we ran these correlation analyses for (ii) the drought/recovery experiment from the three sampling dates in the ambient and “future environment scenario” plots, across both nondrought and drought plots (Figure 1) (“drought subset”), and finally (iii) across all data.

## 3 RESULTS

### 3.1 Effect of temperature and CO_2_ across seasons

Elevated temperature (quadratic term, *p* = .0112) and the interaction between eCO_2_ and eT (*p* = .0110) showed a significant effect on free amino acid concentrations (Table 1). Warming at +1.5°C had a positive effect on free amino acids, while the +3°C temperature had little impact, except for a positive effect (increased by 2.7%) when combined with +300 ppm CO_2_ in May (Figure S4A). We also found a significant effect of season on the free amino acid pool (*p* < .0001), with highest values in May (7.9 ± 1.3 µg N g^−1^ dm, mean ± SD), and decreasing concentrations as the season progressed, with lower concentrations in July (4.1 ± 0.4 µg N g^−1^ dm) and October 2017 (3.7 ± 0.5 µg N g^−1^ dm).

**FIGURE 2:**
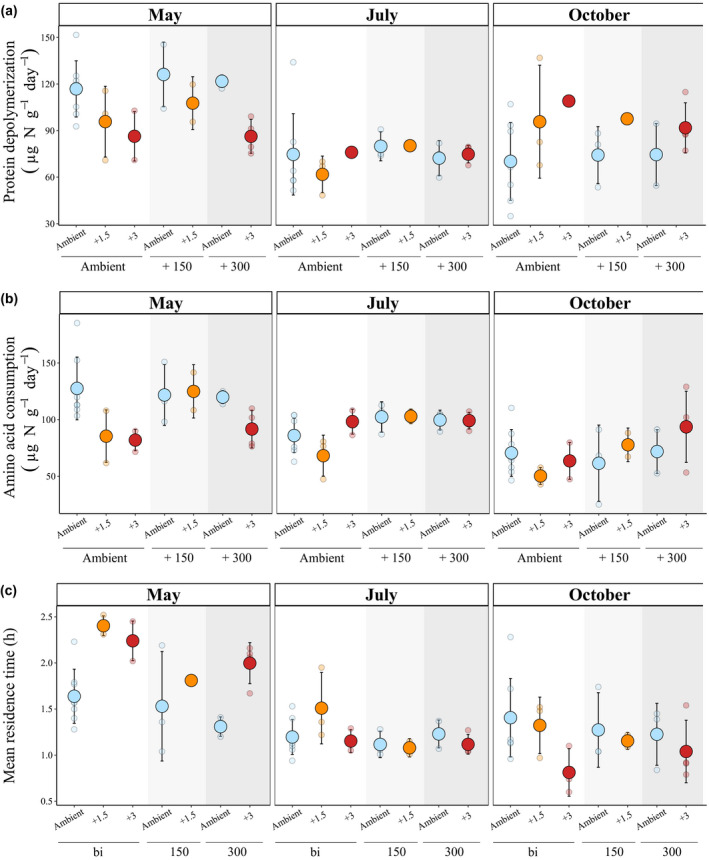
Response of soil organic nitrogen processes to elevated temperature and atmospheric CO_2_ concentration. (a) Protein depolymerization (μg N g^−1^ d^−1^), (b) amino acid consumption (μg N g^−1^ d^−1^), and (c) mean residence times of free amino acids (h) in May, July, and October 2017 under various combinations of three temperatures and three CO_2_ treatment levels. Data points correspond to ambient air temperature (ambient, *blue*), 1.5°C above ambient temperature (+1.5, *orange*), 3°C above ambient air temperature (+3, *red*) within levels of ambient atmospheric CO_2_ concentration (ambient, *white box*), 150 ppm CO_2_ above ambient level (+150, *light gray box*), and 300 ppm CO_2_ above ambient (+300, *dark gray box*). Data are presented as mean ± 1 standard deviation (*n* = 2–8 per treatment, for details see Figure S2), along with raw data (semi‐transparent points). Statistical results of the corresponding generalized least squares models can be found in Table 1. Data for free amino acids, mineralization, and nitrification rates are presented in Figure S4

In general, we found a positive correlation (ρ = 0.724, *p* < .0001) between gross protein depolymerization and microbial amino acid consumption rates (Figure S5). While warming had a marginally significant negative effect on protein depolymerization (*p* = .0630), eCO_2_ significantly increased amino acid consumption rates (Table 1, Figure 2a,b). For example, at ambient temperature, +300 ppm elevated CO_2_ increased amino acid consumption rates on average by 2.5% compared to ambient CO_2_ conditions. There was also a strong effect of the sampling season on the warming effect, on both protein depolymerization and amino acid consumption (*p* < .0001), revealing a significant interactive effect between season and temperature (protein depolymerization *p* < .0001 and amino acid consumption *p* = .0004). The rates of protein depolymerization decreased by 26% in May 2017 (from ambient to +3°C, under ambient CO_2_), were unaffected in August, but responded positively to soil warming (increased by 55%) in October 2017 (Figure 2a). The mean residence time of free amino acids were highest and most variable in May, ranging from 1.04 to 2.52 h, were more uniform across treatments in July, and then responded differently in October (Figure 2c). Mean residence times tended to decrease with increasing CO_2_ (*p* = .0705), but clearly decreased as temperature increased (*p* = .0359, Table 1, Figure 2c). Besides, there was a strong effect of sampling season (*p* < .0001) and of the interaction between season and temperature (*p *< .0001) on mean residence times of amino acids (Table 1). This interactive effect was opposite to the effect on protein depolymerization and amino acid consumption, that is when temperature accelerated these process rates, the mean residence time of amino acids decreased.

Considering inorganic soil N transformation rates, organic N mineralization was not significantly influenced by season (Table 1). There was a significant effect of temperature (*p* = .0490), but this effect was modulated by CO_2_ (*p* = .0006): under ambient CO_2_ levels, warming decreased mineralization rates, especially in the +3°C treatment (Figure S4B). At the +300 ppm CO_2_ level, warming had little (nonsignificantly positive) effect. For nitrification rates, the large range and variation of nitrification rates led to nonsignificant effects of the CO_2_ and temperature treatments (Table 1). However, there was a significant effect of season (*p* < .0001), with rates increasing throughout the growing season (Figure S4C).

### 3.2 Effect of drought and rewetting

**FIGURE 3:**
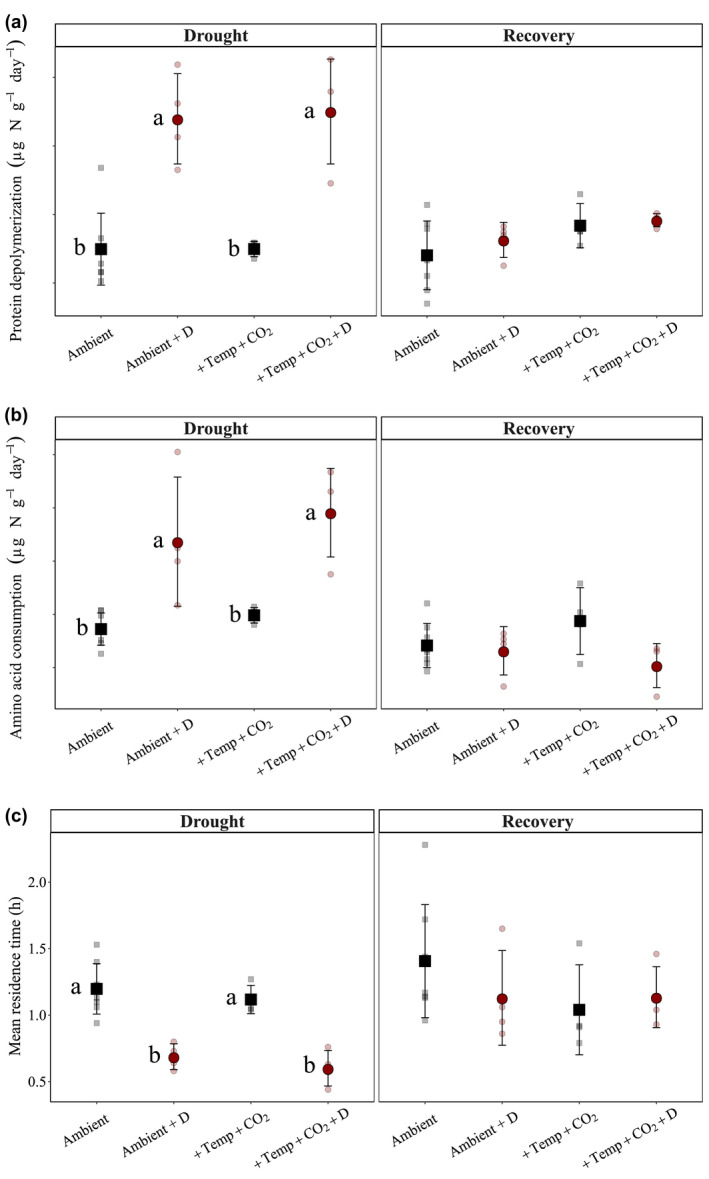
Drought and recovery effects on (a) protein depolymerization (μg N g^−1^ d^−1^), (b) amino acid consumption (μg N g^−1^ d^−1^), and (c) mean residence times of free amino acids (h) in ambient and future climate (+3°C, +300 ppm) plots. Drought effects were measured in July and recovery effects in October 2017 in the “+D” plots (*red points*). Data are presented as mean ± 1 standard deviation (*n* = 4–8 per treatment, for details see Figure S2), along with raw data (semi‐transparent points). Statistical results of two‐way ANOVAs for each variable can be found in Table S2. Points associated with no common letters (Piepho, 2018) are significantly different between groups (*p* < .05, Tukey's HSD test). Data for free amino acids, mineralization, and nitrification rates are presented in Figure S6

Results from the drought‐onset sampling date (May) revealed no significant difference in N process rates and pool sizes for predrought plots (Table S2). The free amino acid pool size was only slightly increased under drought (*p* = .0712) at the peak‐drought sampling in July, in both the ambient and “future scenario” (+3°C and +300 ppm) plots. However, we found twofold increases in both protein depolymerization and amino acid consumption rates in response to drought (*p* < .0001 for both), and an increase in amino acid consumption rates in response to the “future scenario” treatment in July (*p* = .0157) (Figure 3a,b). Drought increased protein depolymerization rates under ambient conditions by 127%, and by 134% under “future scenario” conditions; there was no significant interactive effect between the two, that is, the “future scenario” did not lower or amplify the drought effect on the process rates (Table S2). This was corroborated by Tukey HSD pairwise comparisons, which revealed significant differences in process rates between the drought plots and their paired controls, but no difference between the ambient and “future scenario” plots for protein depolymerization (Figure 3a). We also found twofold decreases in mean residence times in both the drought ambient (by 43%) and the drought “future scenario” (by 46%) climate plots (Figure 3c). For inorganic N transformation processes, drought did not have a significant effect on organic N mineralization in either the ambient or the “future scenario” plots, and rates tended to slightly increase after rewetting (Figure S6). Nitrification rates significantly increased with drought, under both ambient and “future” scenarios (Figure S6).

Three months after rewetting (i.e., at the October sampling date), free amino acid pool sizes were lower in the previously drought‐treated plots (*p* = .0445), but protein depolymerization, amino acid consumption rates, mean residence times of amino acids, as well as mineralization and nitrification rates showed no difference to ambient moisture or nondrought plots (Figure 3). The “future scenario” treatment (+3°C, +300 ppm) and its interaction with drought also showed nonsignificant responses, that is both drought‐treated climate treatments (ambient and “future scenario”) recovered similarly from drought. There was thus no significant legacy effect of drought on either protein depolymerization or amino acid consumption rates, nor on the mean residence time of free amino acids, and mineralization and nitrification rates.

### 3.3 Controls of soil protein depolymerization

Across the entire dataset, we found a strong negative correlation between soil water content (SWC) and gross protein depolymerization (*n* = 102, ρ = −0.710, *p* < .0001) (Figure S7) and amino acid consumption rates (ρ = −0.558, *p* < .0001). Season had a strong effect on SWC. Excluding the drought treatment, which had the lowest SWC of all, the soils were driest in May (0.10–0.25 g H_2_O g^−1^ dm), wettest in July (0.31–0.36 g H_2_O g^−1^ dm), and intermediate in soil water content during October (0.22–0.34 g H_2_O g^−1^ dm).

**FIGURE 4:**
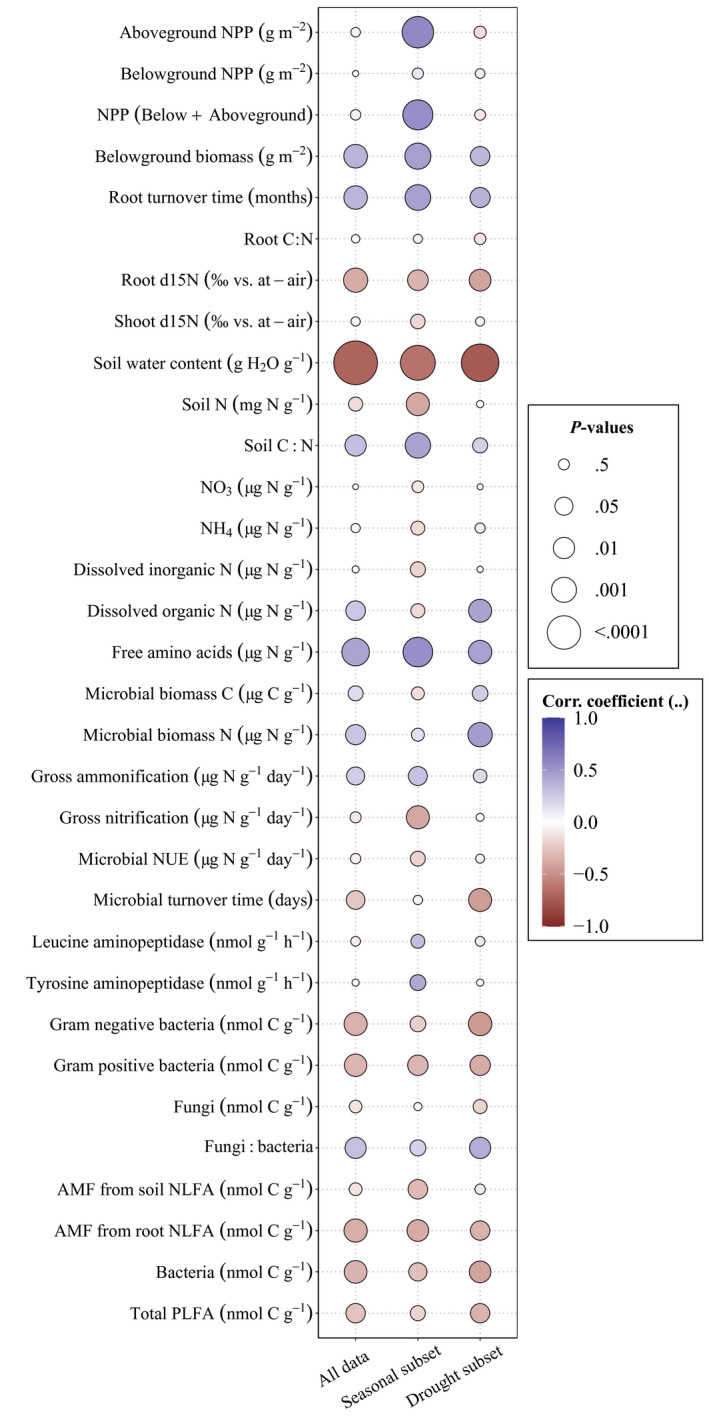
Relationship between protein depolymerization and other parameters, including plant and microbial descriptors, pool sizes, and enzyme activities. The correlation coefficients (ρ) and *p*‐values come from a repeated measures correlation, done for the entire dataset (*n *= 102), for the “eT eCO_2_” subset (ambient, eT, and eCO_2_ plots across seasons, *n* = 78), and for the “drought” experiment subset (ambient and “extreme” climate at the predrought, drought, and recovery dates, *n* = 60)

After running repeated measurement correlations of protein depolymerization with several other parameters, different patterns emerged between the “eT eCO_2_” data subset (ambient, eT, and eCO_2_ plots across seasons) and the “drought” data subset (ambient and “future scenario” at the drought onset, peak drought, and postdrought dates) (Figure 4). In the “eT eCO_2_” dataset, there was a strong positive correlation between protein depolymerization and plant parameters, including aboveground net primary productivity (ρ = 0.576, *p* < .0001), belowground biomass (ρ = 0.463, *p* = .0005), and root turnover time (ρ = 0.454, *p* = .0006). There was also a strong negative correlation between protein depolymerization and total soil N (ρ = −0.395, *p* = .0034), but a positive correlation with free amino acids (ρ = 0.541, *p* < .0001), which are released by protein depolymerization. Subsequent process rates such as ammonification and gross nitrification were positively (ρ = 0.293, *p* = .0335) and negatively (ρ = −0.393, *p* = .0036) related to protein depolymerization, respectively.

Focusing on the “drought” data subset, the relationship of protein depolymerization with aboveground net primary productivity was lost (*p* = .3716), while belowground plant biomass and root turnover time had a much smaller, but still significant positive relationship with protein depolymerization. However, in the “drought” dataset, there was a strong positive relationship between protein depolymerization and dissolved organic N (ρ = 0.443, *p* = .0038), and with microbial biomass N (ρ = 0.479, *p* = .0015). We observed a negative relationship between protein depolymerization and several soil microbial parameters, such as microbial turnover time (ρ = −0.443, *p* = .0037), gram‐negative and gram‐positive bacteria (ρ = −0.454, *p* = .0028; ρ = −0.372, *p *= .0165, respectively), and arbuscular mycorrhizal fungi [AMF] biomass measured by root NLFA analysis (ρ = −0.3450, *p* = .0272) and by PLFA markers (ρ = −0.341, *p* = .0290). The relationships between protein depolymerization and these soil parameters were mostly nonsignificant for the “eT eCO_2_” subset, except for gram‐positive bacteria (ρ = −0.329, *p* = .0161) and AMF as analyzed by root NLFA (ρ = −0.373, *p* = .008).

## 4 DISCUSSION

Organic N forms dominate in soil and their conversion into assimilable N represents the major bottle neck in the terrestrial N cycle (Hu et al., 2020; Schimel & Bennett, 2004; Wanek et al., 2010). Nitrogen is an essential element in all terrestrial ecosystems, and critical for the functioning of all living organisms. As a consequence of anthropogenic activities, its cycle is currently subjected to strong changes. Therefore, understanding the nature and the intensity of the responses of organic N processes to environmental change is central to predicting the future of the terrestrial N cycle, including repercussions on plant productivity and climate‐carbon feedbacks. To the best of our knowledge, this is the first time that multiple climate change factors including warming, elevated CO_2_, and drought were manipulated simultaneously to evaluate individual and interactive effects on organic N processes. Our sampling campaign at different time points during the growing season and our experimental layout allowed to evaluate the effect of seasonality and the presence of nonlinear responses to these global change drivers.

### 4.1 Effects of elevated temperature and elevated CO_2_

In line with our first hypothesis (H_1_), we found no significant effect of increasing atmospheric CO_2_ on protein depolymerization in the fourth year of global change treatments. This is similar to the nonsignificant effect after 8 years of elevated CO_2_ on protein depolymerization in a heathland soil, as found by Wild, Ambus, et al. (2018). Elevated CO_2_ is expected to increase total plant biomass (Dieleman et al., 2012; Ogle et al., 2021), which would result in a larger demand of bioavailable N for plant growth. This could potentially lead to progressive N limitation (Liang et al., 2016; Luo et al., 2004), or might stimulate protein depolymerization to provide the needed plant N. However, because our field site is a managed grassland, fertilizer application likely hindered the development of progressive N limitation. Interestingly, we did not observe nonlinear responses of organic or inorganic N process rates to eCO_2_ nor eT. This indicates that the response of the N cycle within the range of tested values in our climate change treatments (up to 3°C and 300 ppm above ambient) does not reach a plateau and does not change in the direction of effects (i.e., there was no quadratic effect).

We also hypothesized (H_2_) that due to an increase in enzyme activity with increasing temperature, there would be a significant positive effect of eT on protein depolymerization and microbial amino acid consumption rates. We did not find an overall significant positive effect of warming on the process rates, which were measured at in situ field temperatures, but found a highly significant interactive effect between season and temperature (Table 1) on organic N cycling processes, as well as on organic N mineralization. This indicates that the limiting factor acting on soil organic N cycling processes changed between seasons, which modulated or even inversed the effect of warming. Specifically, we found decreased values of protein depolymerization and microbial amino acid consumption during spring, no changes in summer, and increased values in October in warmed plots, compared to control plots (Figure 2). These trends were similar but less marked for mineralization rates. Nitrification, on the other hand, did not reveal any effect of temperature, but the rates increased throughout the growing season. This could be because of the decreasing plant inorganic N demand from spring toward autumn, releasing nitrifiers from substrate (ammonium) competition.

We explain the temperature trends on soil organic N processes as a combinatorial effect of seasonal average temperatures and of substrate availability for proteolytic processes. First, average temperatures are lowest in fall, representing a stronger limiting factor to process rates than in the other seasons. Second, plants generally stop growing and senesce in fall, which increases proteins available for depolymerization via root death (Brunner et al., 2015). At our site, aboveground net primary productivity and belowground biomass were higher in the warmed plots compared to the ambient plots in fall (unpublished data). This is similar to results presented in a meta‐analysis on the effect of combined warming and CO_2_ treatments, which found that on average aboveground biomass increased by 15% and root biomass by 40% in warming treatments (Dieleman et al., 2012; Song et al., 2019). This increase in biomass would thus result in higher plant N uptake, stimulating protein depolymerization in the warmed plots (Fitter et al., 1999) in fall, and explaining the positive effect of temperature at this time of the year. The reason we still found a negative effect of warming on mineralization rates in fall is likely due to an increase in microbial N constraints in fall in warmed plots, causing an increase in microbial NUE and a decrease in microbial ammonium secretion (N mineralization), at a time, when microbial biomass starts to build up during fall and winter. The negative effect of warming on protein depolymerization and mineralization rates in spring may in contrast be due to the earlier onset of plant growth in the warmed plots. Warming was put into effect when snow depth reached <10 cm, which may have stimulated snow melt and triggered an earlier onset of plant growth, as shown before (Leblans et al., 2017). We predict that during the onset of plant growth, a fast activation of depolymerization and mineralization activity quickly consumed available substrate. Due to an earlier onset of plant growth in warmed plots, protein depolymerization rates might have already decreased at the time of measurement due to faster substrate depletion and subsequent substrate limitation of protein depolymerization in contrast to ambient plots. This would also explain why in summer protein depolymerization rates were similar across all plots, when average temperatures are higher and therefore temperature likely does not represent a limiting factor for metabolic processes.

Finally, in accordance with our interaction hypotheses (H_4_), we found no significant interaction effect between warming and elevated CO_2_ on soil organic N processes. This indicates that the effects of warming were not modulated by elevated CO_2_. However, we did find a significant interactive effect between these two global change drivers on gross mineralization rates (Table 1). Under ambient CO_2_, the +3°C treatment decreased rates, while at +300 ppm, the rates were similar between the ambient and +3°C temperature treatments. This significant interactive effect may be explained again due to changes in microbial NUE, though the exact nature of the interactive effect on N mineralization remains prone to speculation.

### 4.2 Effect of drought and rewetting

We further hypothesized (H_3_) that water‐limited conditions during drought would decrease proteolytic activity due to diffusion limitations for soil enzymes and substrates, and thus have a negative effect on protein depolymerization and microbial amino acid consumption. Our results showed the opposite trend: soils collected at the end of the drought period had significantly higher gross rates of protein depolymerization and microbial amino acid consumption under both ambient and “future scenario” climate (Figure 3). This supports the findings by Fuchslueger et al. (2019), who found that summer drought increased protein depolymerization rates in both an extensively managed and in an abandoned subalpine grassland. These results might be explained by the fact that soil microbes can remain hydrated in microsites despite of low SWC (Harris et al., 2021; Homyak et al., 2017), while the osmotic stress associated with drought potentially allows accumulation of microbial N‐rich compounds (Schimel, 2018) at these microsites. Indeed, a positive correlation between microbial biomass N and protein depolymerization rates was found (Figure 4). On the other hand, we found a negative correlation with some of the PLFA biomarkers, which were found to be lower in drought plots. We speculate that the lower PLFA values indicate losses of viable biomass, while the positive correlation with overall microbial biomass C and N suggest that microorganisms accumulated organic compounds within their cells during drought. Microbial death in response to drought might have released protein‐rich cellular contents into the soil and increased protein availability to stimulate protease and peptidase activities. This was not accompanied by an increase in gross N mineralization, likely because of increased N demand and increased microbial NUE. However, nitrification rates also strongly positively responded to drought, likely because of relaxation of substrate (ammonium) limitation of nitrifiers due to strong decreases in plant inorganic N demand under drought. Nevertheless, caution should be exercised when interpreting the results of the IPD approach, as the addition of even a small liquid quantity of ^15^N‐labeled amino acids introduces short‐term/instantaneous rewetting effects (in dry soil from drought plots). The response of protein depolymerization to drought therefore might reflect the immediate increase in protein availability after drought relaxation (~60‐min response time).

Two months after rewetting, no legacy effect of the drought was observed (Figure 3) and protein depolymerization returned to rates similar to undisturbed plots. This is linked to the recovery of the soil microbial community, whose respiration and growth also fully recovered after the rewetting event (Simon et al., 2020). The soil inorganic N processes, that is N mineralization and nitrification, also recovered to predrought levels, as previously documented (Fuchslueger et al., 2014).

### 4.3 Drivers of protein depolymerization and indications of substrate limitation

When correlating protein depolymerization with other potential drivers in the “eT eCO_2_” dataset (ambient conditions, eT, and eCO_2_), we observed multiple positive correlations between protein depolymerization and plant‐related variables, such as ANPP, net primary productivity (NPP), belowground biomass, and root turnover time (Figure 4). This suggests that microbial N limitation triggers an increased allocation of resources toward soil organic N mining and therefore protein depolymerization, when plant N uptake or rhizodeposition and priming processes prevail in the system. Indeed we found higher protein depolymerization rates with high root turnover time (slow root turnover rate), low root and shoot δ^15^N (proxies for a more conservative ecosystem N cycling) (Robinson, 2001), and low soil total N and thus high soil C:N (Zechmeister‐Boltenstern et al., 2015).

When focusing on the “drought” dataset (ambient versus “future scenario,” ± drought), we found a strong shift in the explanatory variables of protein depolymerization rates compared to the “eT eCO_2_” dataset. The positive correlation between protein depolymerization and ANPP from the “eT eCO_2_” dataset was lost and the positive relationship with belowground biomass and root turnover time became markedly weaker (Figure 4). During drought conditions, gross primary production and plant biomass is reduced along with C input to soil (Meeran et al., 2021). Instead, many relationships with microbial‐related parameters became more significant. We suggest that this indicates a shift from plant control in the “eT eCO_2_” subset to soil microbial substrate control, accelerating protein depolymerization during drought/rewetting. Specifically, protein depolymerization was negatively correlated with many PLFA biomarkers, which represent viable microbial biomass markers and therefore suggest that losses of active microbial biomass increase protein depolymerization rates. Indeed, microbial residues (i.e., the depolymerization of microbial cell walls) were shown to trigger the N cycle (Hu et al., 2020). On the contrary, we found a positive relation between microbial biomass C and N with protein depolymerization, which suggests potential accumulation of internal compatible solutes during drought, potentially in the form of C‐ and N‐rich osmolytes. This may be plausible given that such drought adaptation strategies of soil microbial communities have been previously observed (Malik et al., 2020; Schimel, 2018; Warren, 2014).

Soil protein depolymerization is not subject to direct metabolic control and can either be enzyme or substrate limited (Mooshammer et al., 2012; Noll, Zhang, & Wanek, 2019). In the drought‐treated plots, we observed lower potential leucine and tyrosine aminopeptidase activities (Canarini et al., in preparation). Nevertheless, gross protein depolymerization rates were higher in the drought plots. Therefore, we conclude that protein depolymerization was not enzyme limited but rather substrate limited, and this substrate limitation was relaxed under drought conditions due to large inputs of proteolytic substrates, as found in previous studies (Geisseler & Horwath, 2008; Noll, Zhang, & Wanek, 2019). The indication that proteolytic enzyme activity is controlled by protein supply to proteases is also supported by further evidence. First, throughout the growing season, the range of protein depolymerization values varied little compared to the large variance of potential N‐related enzyme activities. Besides, we observed high rates of protein depolymerization under low soil N, low root and shoot δ^15^N, and high soil C:N (Figure 4). All four parameters, which are positively correlated with protein depolymerization, are proxies for microbial and plant N limitation and indicate that protein depolymerization increases when plants and microbes show a conservative N cycling in grasslands, thereby highlighting that protein depolymerization is demand driven. Substrate limitation of protein depolymerization rates rather than enzyme limitation may also partially explain why we did not find an overall positive effect of warming on organic N process rates (protein depolymerization and microbial amino acid consumption), as the seasonal changes in protein availability may have constrained an overall positive temperature effect (Brzostek & Finzi, 2011; Davidson & Janssens, 2006).

Finally, our study provides evidence in support to the notion that the depolymerization of N‐containing organic polymers represents the bottleneck in the soil N cycle (Hu et al., 2020; Jan et al., 2009; Schimel & Bennett, 2004; Wanek et al., 2010). The values of protein depolymerization exceeded those of gross rates of organic N mineralization and nitrification measured here during the same sampling campaigns at the experimental site, by 10‐ to 30‐fold (Figure S4). These 10‐ to 30‐fold higher rates of protein depolymerization indicate that these rates are the limiting step within the measured N cycle and that amino acid and oligopeptide availability is not sufficient to support high mineralization and nitrification rates. If N limitation triggers N mining by protein depolymerization, the subsequent inorganic N cycling processes using excess N (nitrification, nitrate consumption by denitrifiers, etc.) may decrease.

## 5 CONCLUSIONS

In our study, we demonstrate a strong response of organic N cycling to multiple global change factors and a strong modulating role of seasonality. We show a shift in the control of soil protein depolymerization, from plant substrate availability under continuous environmental change drivers (warming and elevated CO_2_) to microbial turnover and soil organic N availability under the pulse disturbance of a drought event. Elevated CO_2_ showed no individual effect, likely due to currently lacking responses of plant biomass production at our site. In contrast, plant biomass production increased in warmed plots and showed a strong correlation with soil organic N processes, whereas drought effects showed significant correlations with microbial‐related parameters. We also observed that the effects of eT on microbial‐driven N processes were modulated by season, which we attribute to a combination of changes in substrate availability and average seasonal temperature. Seasonality, via shifts in the limiting factors controlling soil organic N processes, acts as a strong determinant of climate change effects. Finally, our data indicate that protein depolymerization is the key process in soil N cycling and that it is mostly substrate limited. To the best of our knowledge, this is the first study analyzing the effects of multiple global change factors and levels, and of seasonality on soil organic N cycling. Given the greater implications of the N cycle for N losses and climate feedbacks, understanding how different climate change scenarios impact soil organic N processes represents an invaluable information to predict global change effects on terrestrial N cycling.

## Supporting information

Supplementary Material

## Data Availability

The data that support the findings of this study are openly available in Zenodo at http://doi.org/10.5281/zenodo.5597021.
