# Supplementary material for: Contrasting drivers of belowground nitrogen cycling in a montane grassland exposed to a multifactorial global change experiment with elevated CO2, warming, and drought
Source: Glob Chang Biol. 2022 Jan 10;28(7):2425–41. doi: 10.1111/gcb.16035 (PMC9306501; doi:10.1111/gcb.16035)
Supplement: Supplementary file 1 — Supplementary Material [file GCB-28-2425-s001.docx]

# Supplementary Tables & Figures

**Table S1:** Incubation temperatures for the isotope pool dilution measurements according to each of the field temperature treatments (the mean of the three replicates per temperature treatment) in May, July, and October 2017.

| **Temperature treatment** | **May** | **July** | **October** |
| --- | --- | --- | --- |
| ambient | 16.0 | 17.0 | 13.0 |
| + 1.5 °C | 17.5 | 18.5 | 14.5 |
| + 3 °C | 19.0 | 20.0 | 16.0 |

**Table S2:** Statistical results of two-way ANOVAs for each sampling season, shown separately for each season, testing the effect of climate (ambient = + 0 ⁰C & + 0 ppm CO_2_ vs ‘future’ = + 3 ⁰C & + 300 ppm CO_2_), drought, and their interaction, on free amino acid pool size, protein depolymerization, amino acid consumption and mean residence time of amino acids. P-values below 0.05 are in bold and represent significant results, with the limit set to alpha=0.05.

|  | **Free amino acids** | | **Protein depolymerization** | | **Amino acid consumption** | | **Mean residence time** | | **Mineralization** | | **Nitrification** | |
| --- | --- | --- | --- | --- | --- | --- | --- | --- | --- | --- | --- | --- |
|  | F-value | p-value | F-value | p-value | F-value | p-value | F-value | p-value | F-value | p-value | F-value | p-value |
| **May** | | | | | | | | | | | | |
| climate | 0.0504 | 0.8253 | 3.6715 | 0.0734 | 5.0486 | **0.0391** | 6.7153 | **0.0197** | 1.8940 | 0.1877 | 0.2424 | 0.6291 |
| drought | 0.0966 | 0.7599 | 0.0548 | 0.8179 | 3.4636 | 0.0812 | 0.5775 | 0.4583 | 6.3079 | **0.0231** | 0.7348 | 0.4040 |
| climate:drought | 3.5642 | 0.0773 | 3.9184 | 0.0652 | 4.0136 | 0.0624 | 0.6536 | 0.4307 | 1.2927 | 0.2723 | 0.3078 | 0.5867 |
| **July** | | | | | | | | | | | | |
| climate | 0.1123 | 0.7419 | 2.1099 | 0.1657 | 7.3043 | **0.0157** | 7.2489 | **0.016** | 7.0844 | **0.0171** | 0.9529 | 0.3435 |
| drought | 3.735 | 0.0712 | 56.4066 | **<0.0001** | 40.6525 | **<0.0001** | 63.1312 | **<0.0001** | 2.9775 | 0.1037 | 36.9418 | **<0.0001** |
| climate:drought | 0.2929 | 0.5958 | 0.0398 | 0.8444 | 0.0144 | 0.9058 | 0.3296 | 0.5739 | 0.2312 | 0.6371 | 4.9893 | **0.0401** |
| **October** | | | | | | | | | | | | |
| climate | 0.1373 | 0.7159 | 5.2864 | **0.0353** | 0.0045 | 0.9471 | 2.0405 | 0.1724 | 1.7150 | 0.2088 | 1.6474 | 0.2176 |
| drought | 4.753 | **0.0445** | 0.8627 | 0.3668 | 3.4047 | 0.0836 | 0.3884 | 0.5419 | 0.9075 | 0.3549 | 3.5987 | 0.0760 |
| climate:drought | 0.0853 | 0.774 | 0.1675 | 0.6878 | 2.1851 | 0.1588 | 1.6669 | 0.215 | 0.0424 | 0.8394 | 2.2885 | 0.1498 |

**Figure S1.** A brief diagram of the nitrogen cycle in a managed grassland. Adapted from Schimel and Bennett (2004) and Noll et al. (2019).

**
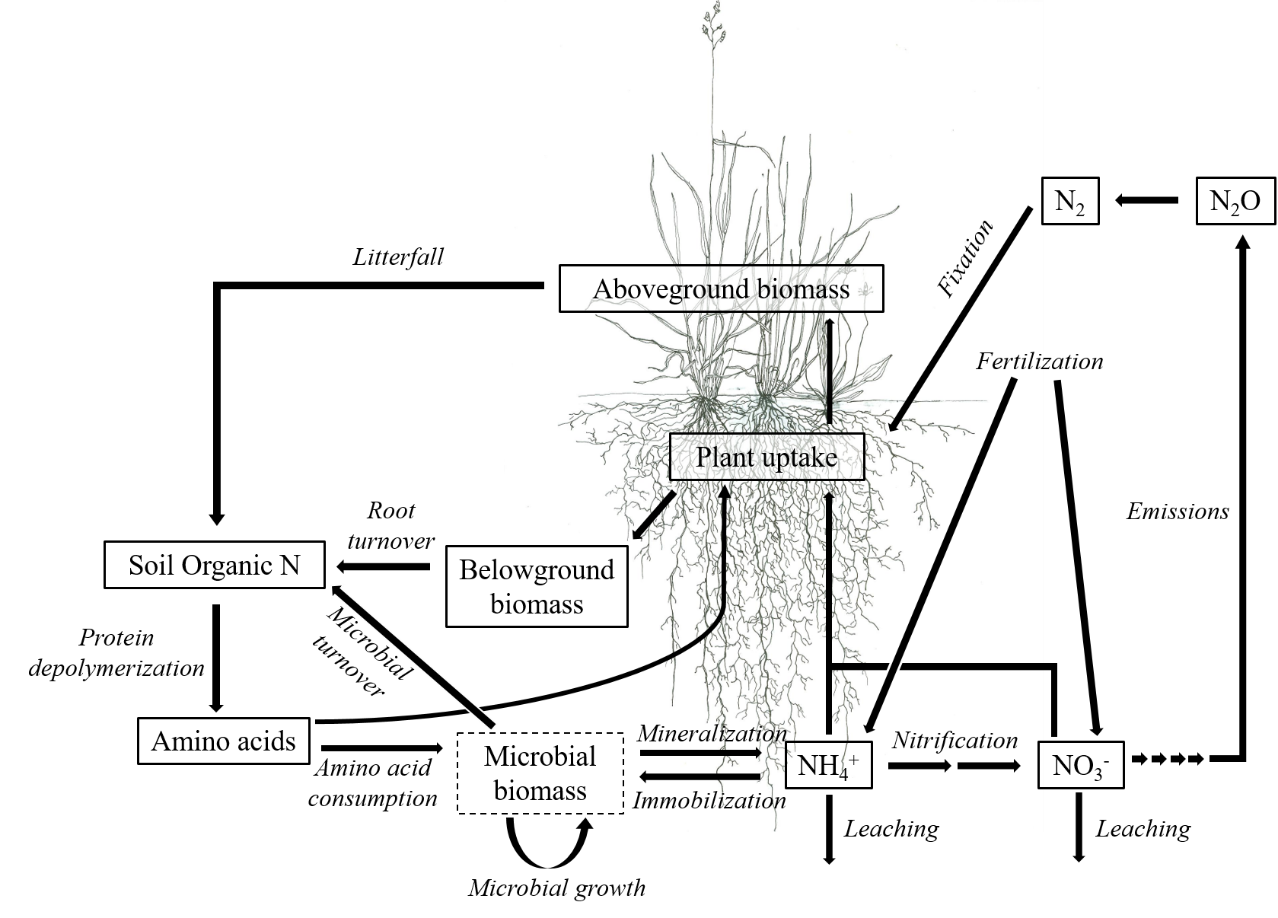
**

**
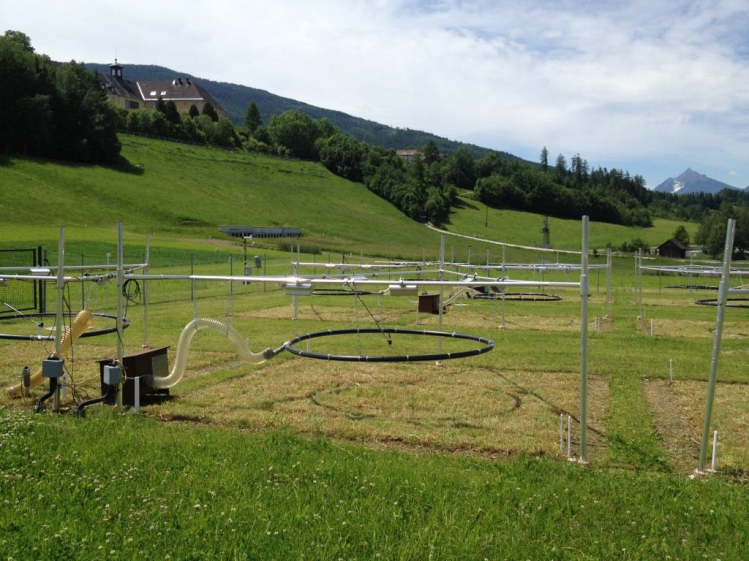
Figure S2.** Experimental T-FACE set-up in Gumpenstein, Austria, picture directly taken after a seasonal cut. Ambient air is blown into the plots through the black circular tubes and enriched with CO_2_ for the eCO_2_ treatments by 150 or 300 ppm. Six infrared heaters are placed on the upper metal rods in each plot as dummies (ambient temperature) or to enhance air temperature in proximity to the vegetation by +1.5 or +3.0 °C.

**Figure S3.** The principle of the isotope pool dilution technique. (A, B) The fraction of ^15^N in amino acids (AA) found in the first incubation, stopped 15 minutes (min) after adding ^15^N-AA, and for the second incubation, stopped after 60 min. (C) The evolution of the ^15^N fraction: the
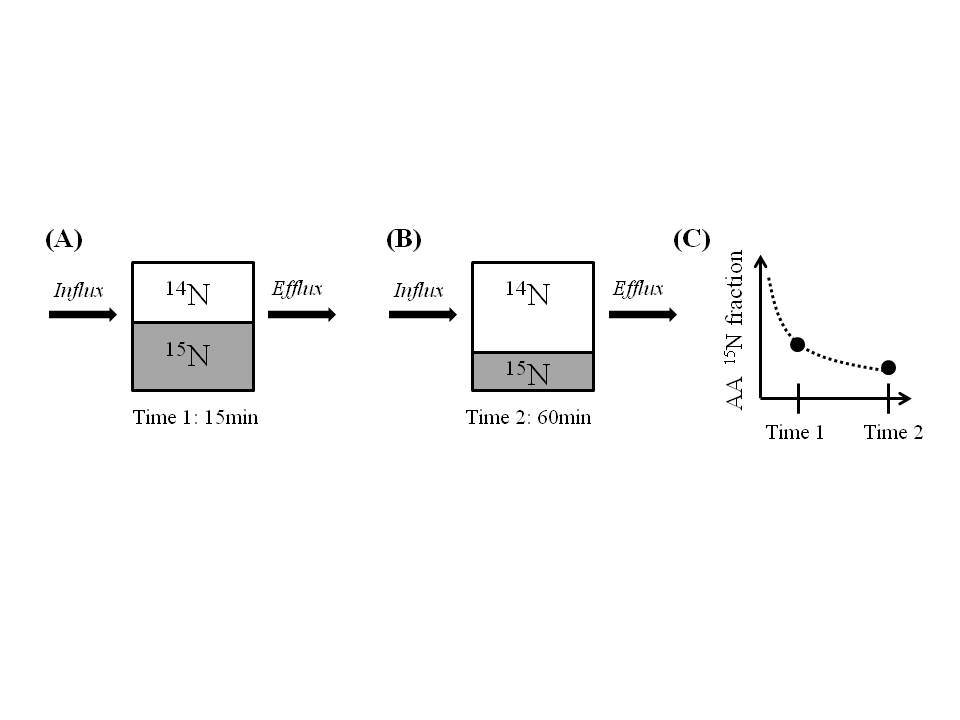
tracer is quickly consumed, and then decreases at a constant rate between the two time points.

**Figure S4.** Response of A) free amino acid pools (μg N g^-1^), B) mineralization (μg N g^-1^ d^-1^), and C) nitrification rates (μg N g^-1^ d^-1^) to elevated temperature and atmospheric CO_2_ concentration in May, July and October 2017 under various combinations of three temperatures and three CO_2_ treatment levels. Data points correspond to ambient air temperature (ambient, *blue*), 1.5 °C above ambient temperature (+1.5, *orange*), 3 °C above ambient air temperature (+3, *red*) within levels of ambient atmospheric CO_2_ concentration (ambient, *white box*), 150 ppm CO_2_ above ambient level (+150, *light grey box*), and 300 ppm CO_2_ above ambient (+300, *dark grey box*). Data are presented as mean ± 1 standard deviation (n = 2-8 per treatment, for details see Fig. S2), along with raw data (semi-transparent points). Statistical results of the corresponding generalized least squares models can be found in Table1.

**
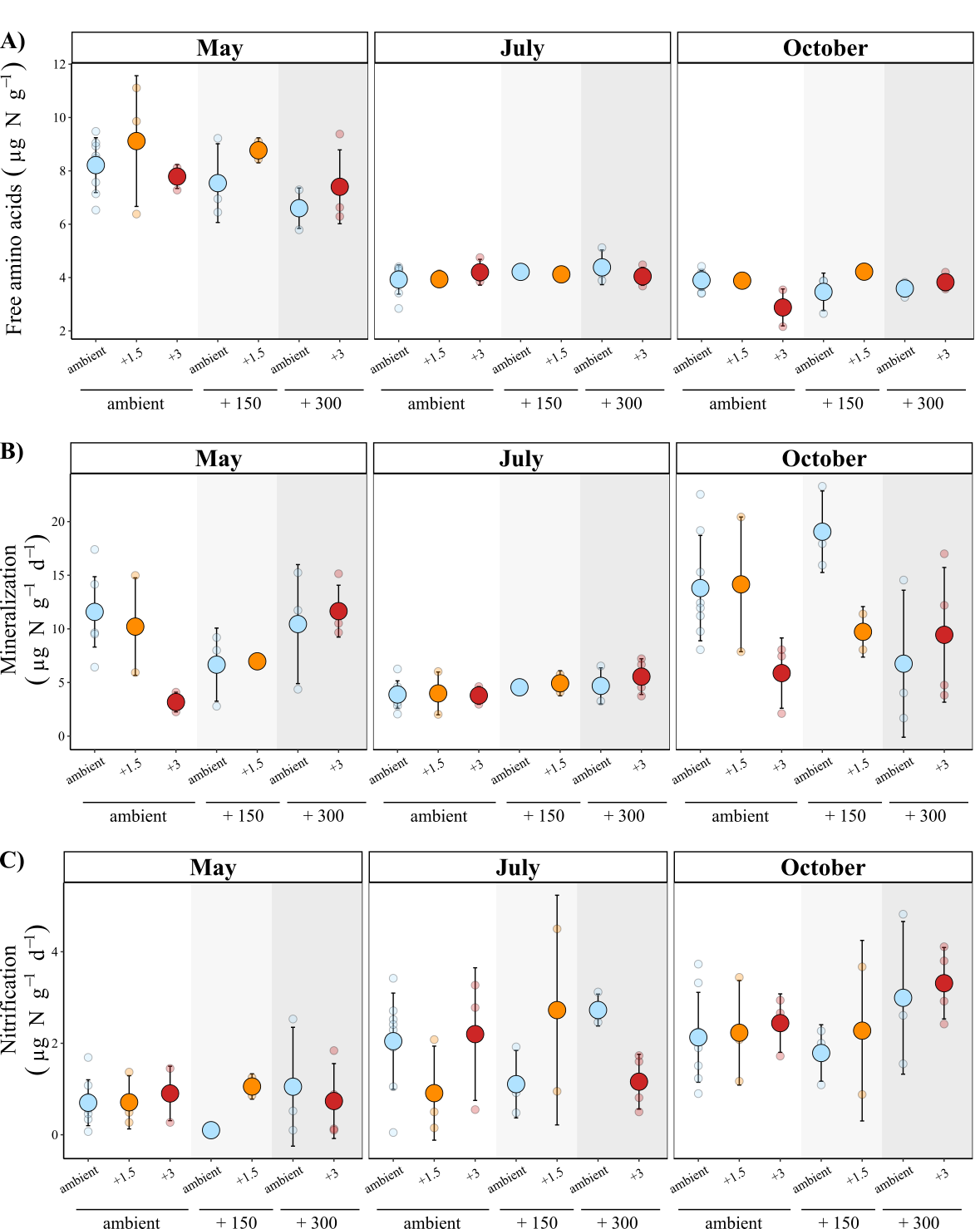
**

**
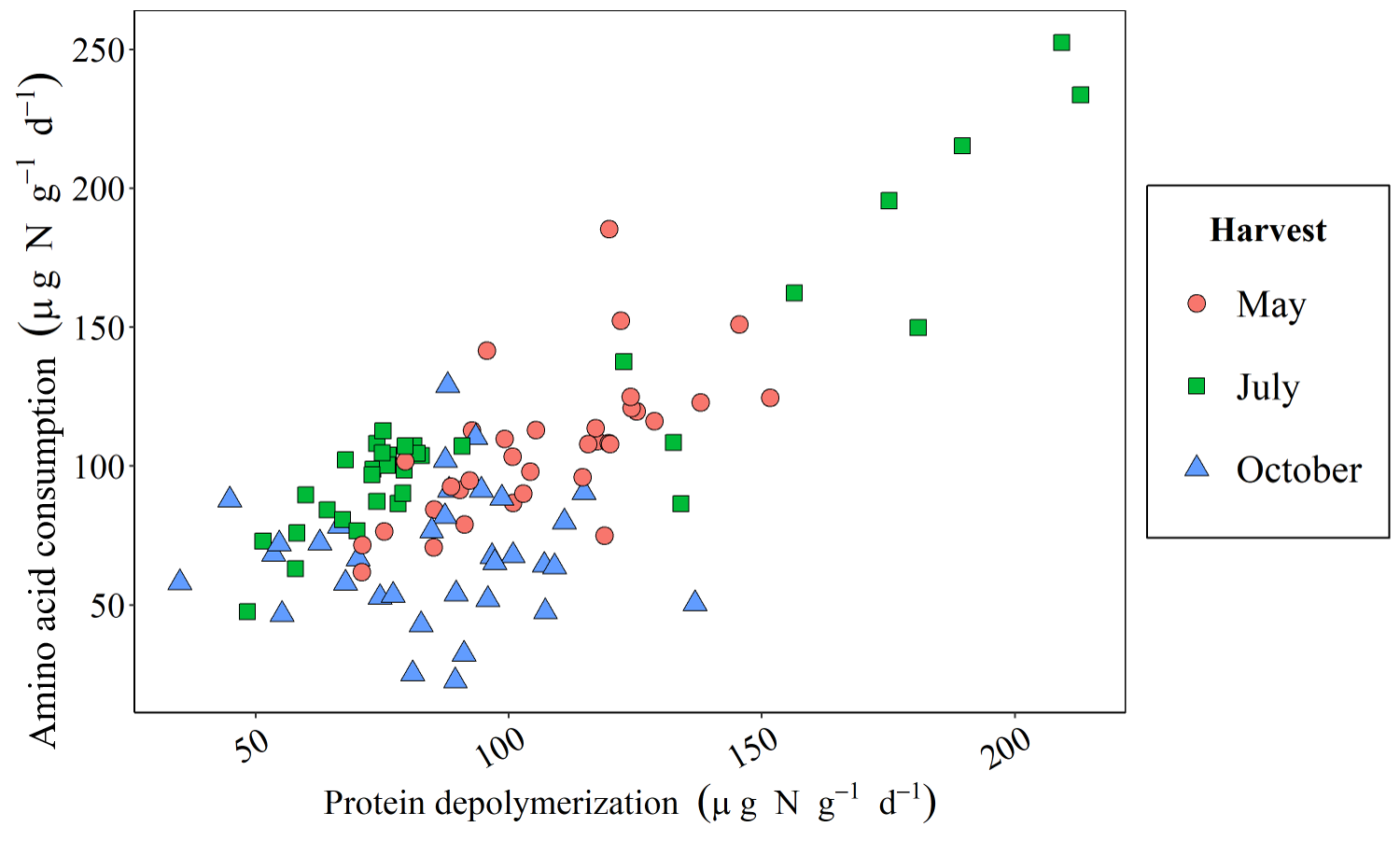
Figure S5.** Positive relationship between gross rates of protein depolymerization and microbial amino acid consumption across seasons and global change treatments. Filled symbols refer to different seasons: ● May, ■ July, and ▲ October 2017 (n =102). ρ corresponds to the correlation coefficient and its associated p-value after running the repeated measures correlation.

ρ = 0.7462

p < 0.0001

**Figure S6.** Drought and recovery effects on A) mineralization (μg N g^-1^ d^-1^) and B) nitrification rates (μg N g^-1^ d^-1^) in ambient and future climate (+ 3° C, +300 ppm) plots. Drought effects were measured in July and recovery effects in October 2017 in the ‘+ D’ plots (red points). Data are presented as mean ± 1 standard deviation (n = 4-8 per treatment, for details see Fig. S2), along with raw data (semi-transparent points). Statistical results of two-way ANOVAs for each variable can be found in Table S2. Points associated with no common letters (Piepho, 2018) are significantly different between groups (p < 0.05, Tukey’s HSD test).

**
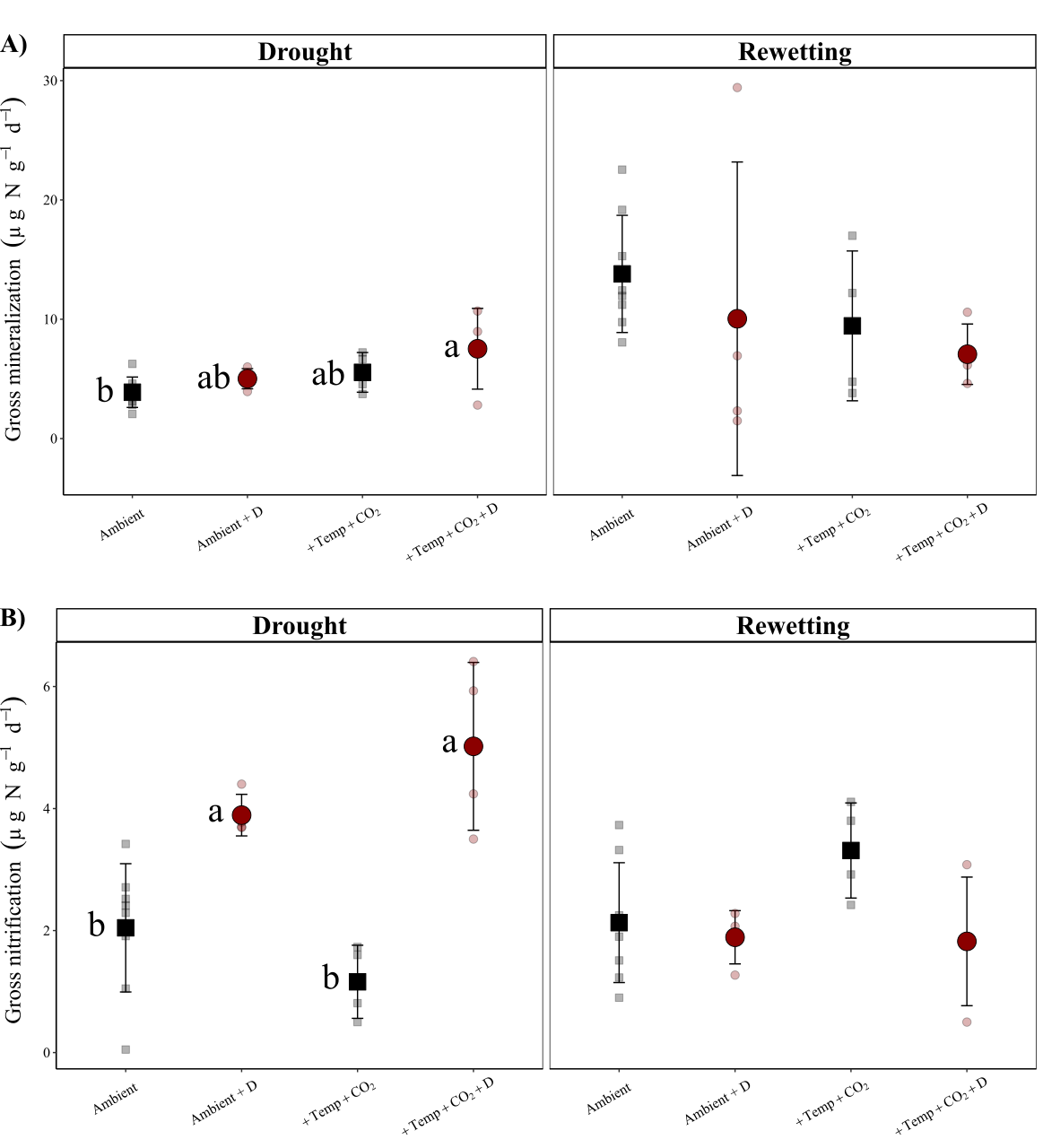
**

**Figure S7.** Relationship of gross protein depolymerization rates with soil water content. Filled symbols refer to different seasons: ● May, ■ July, and ▲ October 2017 (n = 102). The drought-treated plots in July are presented by the ■ symbols in the dashed box. ρ corresponds to the correlation coefficient and its associated p-value after running the repeated measures correlation.


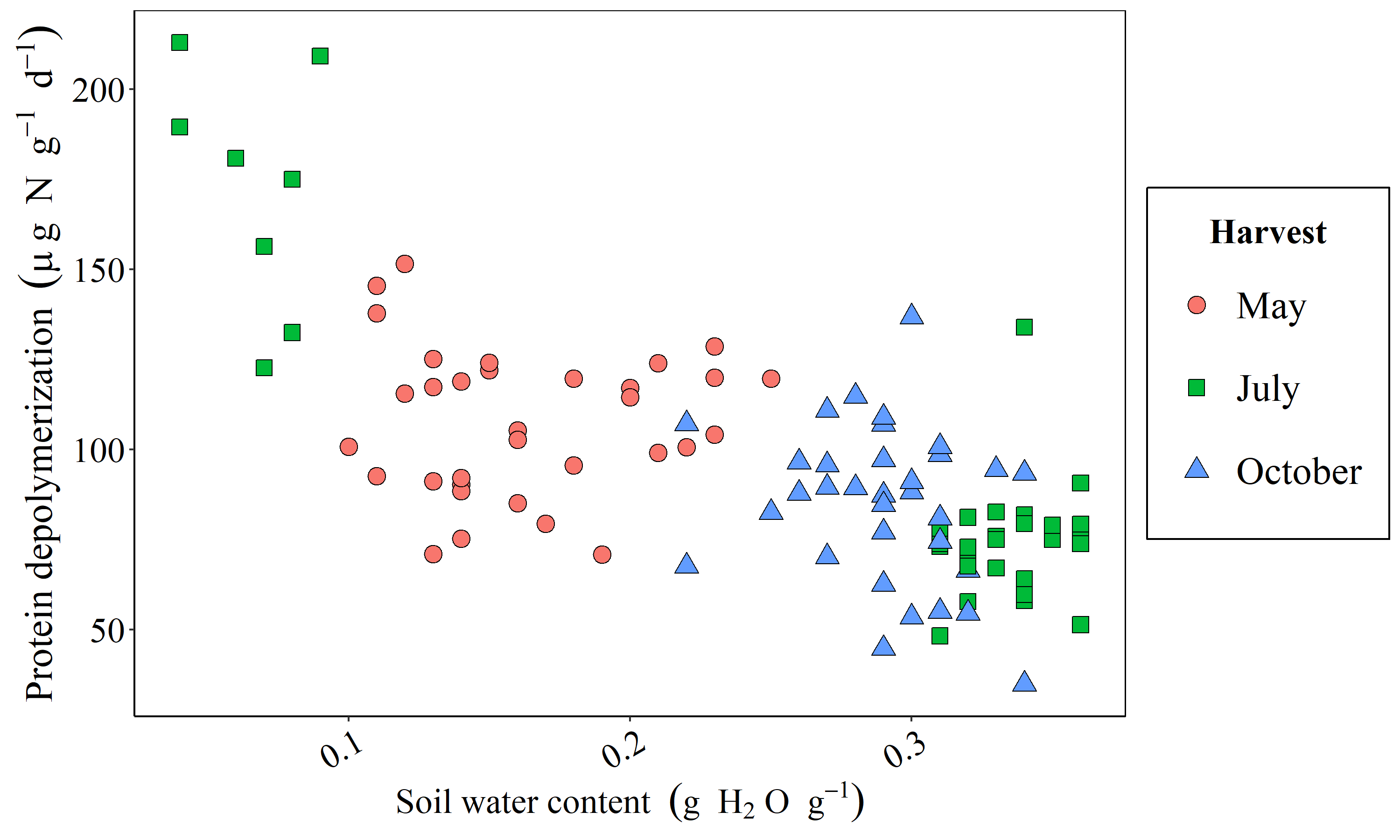


ρ = -0.7095

p < 0.0001

**References**

Noll, L., Zhang, S., Zheng, Q., Hu, Y., Wanek, W., 2019. Wide-spread limitation of soil organic nitrogen transformations by substrate availability and not by extracellular enzyme content. Soil Biology and Biochemistry 133, 37–49. doi:10.1016/j.soilbio.2019.02.016

Piepho, H.-P., 2018. Letters in Mean Comparisons: What They Do and Don’t Mean. Agronomy Journal 110, 431–434. doi:10.2134/agronj2017.10.0580

Schimel, J.P., Bennett, J., 2004. Nitrogen mineralization: challenges of a changing paradigm. Ecology 85, 591–602.
